# Mothers' sociodemographic factors and use of health professionals for child feeding advice

**DOI:** 10.1111/mcn.13586

**Published:** 2023-11-06

**Authors:** Eve House, Huilan Xu, Sarah Taki, Elizabeth Denney‐Wilson, Louise Baur, Li M. Wen

**Affiliations:** ^1^ Sydney School of Public Health, Faculty of Medicine and Health The University of Sydney Sydney NSW Australia; ^2^ NHMRC Centre of Research Excellence in Translating Early Prevention of Obesity in Childhood (EPOCH‐Translate) Sydney NSW Australia; ^3^ Sydney Institute for Women Children and Their Families, Sydney Local Health District Sydney NSW Australia; ^4^ Health Promotion Unit Population Health Research & Evaluation Hub, Sydney Local Health District Sydney NSW Australia; ^5^ Susan Wakil School of Nursing and Midwifery Faculty of Medicine and Health, University of Sydney Sydney NSW Australia; ^6^ Specialty of Child and Adolescent Health Sydney Medical School, Faculty of Medicine and Health, The University of Sydney Sydney NSW Australia

**Keywords:** bottle feeding, breastfeeding, child nutrition sciences, feeding behaviour, health promotion, parenting, primary health care

## Abstract

This study examined sociodemographic factors associated with mothers seeking child feeding advice from health professionals (HPs). Cross‐sectional analysis of survey data from linked randomized controlled trials was conducted. Surveys asked which sources of feeding information mothers used when their child was 6 months and 5 years old. Logistic regression was used to examine associations between sociodemographic characteristics and use of information from HPs. Here, 947 and 405 mothers completed 6‐month and 5‐year surveys, respectively. At 6 months, multiparous mothers were less likely to seek advice from child and family health nurses (CFHNs) (adjusted odds ratio [AOR]: 0.558, 95% confidence interval [95% CI]: 0.416–0.749) and other HPs (AOR: 0.706, 95% CI: 0.542–0.919), unmarried mothers were less likely to seek advice from other HPs (AOR: 0.582, 95% CI: 0.342–0.990). At 5 years, mothers with household income ≥$80,000 p.a. were less likely to seek advice from CFHNs (AOR: 0.514, 95% CI: 0.302–0.875) and working mothers less likely to seek advice from general practitioners (GPs) (AOR: 0.581, 95% CI: 0.374–0.905). Mothers born in Australia were less likely to seek information from CFHNs (AOR: 0.462, 95% CI: 0.257–0.833) and GPs (AOR: 0.431, 95% CI: 0.274–0.677). There was a greater likelihood that multiparous mothers (AOR: 2.114, 95% CI: 1.272–3.516) and mothers of children whose fathers had not attended university (AOR: 2.081, 95% CI: 1.256–3.449) had never sought advice from CFHNs, and that mothers who had not attended university (AOR: 1.769, 95% CI: 1.025–3.051), multiparous (AOR: 1.831, 95% CI: 1.105–3.035) and employed (AOR: 2.058, 95% CI: 1.135–3.733) mothers had never sought advice from other HPs. Understanding sociodemographic factors associated with seeking child feeding advice from HPs may inform priorities for engaging families in health promotion.

## Key messages

- Many mothers seek infant and child feeding advice from primary health professionals, particularly in the first year of life, with engagement declining at age 5 years.
- Health promotion efforts in primary care settings should prioritize increased engagement of multiparous mothers, those without university education and those in employment.
- The internet and peers are important sources of infant and child feeding information for mothers, it is important that evidence‐based information is available to mothers online and through peer‐learning models.

## Graphical abstract

Mothers often seek child feeding information from primary health professionals in early life. Attention should be given to engaging multiparous mothers, those without a university education and employed mothers in preventive care. Evidence‐based feeding information should also be available to mothers online and through peer‐learning models.

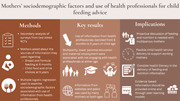

## 1 INTRODUCTION

Across the first 5 years of life, nutrition has a substantial impact on children's development and later health outcomes. In the first 6 months, establishing and maintaining breastfeeding can have significant positive impacts on infant health, reducing both immediate health risks, such as infection, and long‐term health concerns such as obesity and adverse metabolic health outcomes compared with infants receiving infant formula (Dieterich et al., 2013). Furthermore, the use of responsive feeding practices in infancy—whether breastfeeding or bottle feeding—is associated with healthy growth and reduced risk of overweight or obesity in childhood (Redsell et al., 2021). The toddler years are a critical period to influence children's food preferences. From age 1–3 years children establish most of their food preferences. Exposure is a key driver of food preference, as children with greater exposure to fruits and vegetables tend to have higher intakes of these core foods in later childhood (Howard et al., 2012). There is an association between children's food preferences and maternal food preferences, whereby children are more likely to be exposed to and have a preference for foods that their mothers also like (Howard et al., 2012).

Parents and carers act as important nutritional gatekeepers for young children, as they are responsible for most of the decision‐making regarding infant feeding, food purchasing and food preparation. Parental knowledge of infant feeding and nutritional recommendations is essential for the promotion of optimal early childhood nutrition. In a study of first‐time mothers in their third trimester, mothers who were aware of the guidelines for exclusive breastfeeding were more likely to report an intention to breastfeed exclusively for at least 6 months, compared with those who were not aware of the recommendations (Wen et al., 2009). Similarly, when children begin to eat family foods, a combination of high confidence in nutritional knowledge and cooking confidence among primary dietary gatekeepers (i.e., the parents/carers completing the majority of food acquisition and preparation) is associated with healthier food purchasing and preparation practices (Reid et al., 2015). Thus, it is imperative that accurate infant and child feeding and nutrition information is readily accessible to parents of infants and young children.

In Australia, universal child health services are available to families throughout pregnancy and the first 5 years of life. Child and family health nurses (CFHNs) provide well‐being and developmental checks at key stages throughout early childhood at no cost to families, with core components of their service including developmental screening and health promotion (NSW Child and Family Health Nursing Clinical Nurse Consultant Network & Child and Family Health Nurses Association NSW, 2022). In addition, general practitioners (GPs) may be an important source of health information for families in the first 5 years, with previous research, indicating that parents seek health information from a variety of health professionals, dependent upon the type of information they require (Rossiter et al., 2019). Given that primary health professionals have frequent contact with young children throughout early childhood, they are in a promising position to provide parents with advice regarding their child's feeding and nutritional concerns.

It is important to understand the factors that may influence parents' engagement with health professionals for feeding and nutrition advice, to better tailor health services and alternative sources of information to meet their needs. Thus, the primary aims of this study were to identify the sociodemographic factors associated with parents' engagement with health professionals for feeding and nutrition advice, and to identify demographic characteristics of those who never used CFHNs or other health professionals at both 6 months and 5 years of their children's ages. The secondary aim was to describe the sources of information used by parents for children's feeding and nutrition information and their perceived helpfulness.

## 2 METHODS

### 2.1 Study design

Secondary analysis was conducted using data extracted from linked randomized controlled trials (RCTs) (Wen et al., 2017, 2019) conducted in Australia from 2017 to 2022. Data examined in this study were collected at baseline (third trimester) and 6 months of child age in the first RCT (between October 2017 and July 2018), and at 5 years of age in the second RCT (between March 2022 and October 2022). The full study protocols for each RCT have been published elsewhere (Wen et al., 2017, 2019). In short, the first trial was a three‐arm RCT that aimed to examine the efficacy of a telephone (Intervention group 1) or SMS (Intervention group 2) delivered early childhood obesity prevention programme compared with usual care (control group) (Wen et al., 2020, 2022). The intervention was delivered in the antenatal period and the first 12 months of life, with follow‐up until 24 months. At 24 months mothers who remained in the RCT were invited to participate in the second trial and were randomized to receive either a combined phone and SMS intervention (intervention group) or usual care (control group), the intervention was delivered for 24 months, that is, until age 4 years, with follow‐up until age 5 years (Wen et al., 2023). In both trials, the intervention groups received a staged early intervention programme in which key health promotion messages aligned with developmental milestones (e.g., breast and bottle‐feeding addressed at third trimester to 3 months; timing of introduction of solids at 3–5 months; healthy food choices at 12 months; repeated exposures to healthy foods and promoting active play at 2–3 years) based on the principles of the effective *Healthy Beginnings* programme (Wen et al., 2012, 2017, 2019).

### 2.2 Participants

A total of 1155 pregnant women were recruited to the first RCT in 2017 from antenatal clinics of eight hospitals in four local health districts in Sydney, Australia. To be eligible for the study, women needed to be 16 years of age or older, have a mobile phone, be able to communicate in English and live within the catchment area of the eight hospitals. Women with severe medical conditions, expecting multiple births or with known foetal abnormalities were excluded. When children were 2 years of age, 662 mothers consented to participate in the second trial from 2 to 5 years of age.

### 2.3 Data collection and measurement

Data were collected using telephone surveys conducted at baseline, when the child was 6 months old and 5 years old. Surveys were delivered by a marketing survey company using the computer‐assisted telephone interviewing system.

#### 2.3.1 Demographic information

Demographic information was collected at baseline of the first trial. This included mothers' country of birth, marital status, parity, education level and employment status; language spoken at home; household income; and fathers' education level and employment status. Mothers' date of birth was collected during recruitment and maternal age was calculated based on the date of baseline survey completion. Sociodemographic variables were grouped for analysis (Wen et al., 2020).

#### 2.3.2 Sources of nutrition information

Items regarding parental sources of nutrition information were adapted from the baseline survey used in the first trial (Wen et al., 2020) and from a previous study (Denney‐Wilson et al., 2015). Questions asked mothers to identify the sources they used to obtain information about breast or formula feeding when their child was 6 months of age, and children's food and drink choices when their child was 5 years of age. Sources included friends and family, health professionals, telephone helplines, social media, apps, websites, educators and the ‘Blue Book’, which is a personal health record book provided to parents of every child born in New South Wales (NSW), the most populous state in Australia (NSW Ministry of Health, 2021). At 6 months, mothers in the intervention groups could also indicate whether they used the intervention material (i.e., *Healthy Beginnings*) for breast and formula feeding information, this data is not presented in this analysis. For each source, mothers were asked to indicate ‘yes’ or ‘no’, as to whether they used the source. Of those sources used, mothers were asked to rate the helpfulness of each source on a 3‐point Likert Scale, ‘Very helpful’, ‘Somewhat helpful’ or ‘Not helpful at all’. Mothers were then asked to identify the source of information they used the most often at each time point (see Appendix 1 for full survey items). Mothers' use of health professionals over time was split into two categories—‘never used’ or ‘ever used’. For the analysis of the use of health professionals over time, at 5 years the use of ‘other health professionals’ included the combined use of GPs, paediatricians, dietitians and other health professionals.

### 2.4 Statistical analysis

Statistical analysis was conducted in IBM SPSS Statistics^©^ (V29.0.0). Descriptive analysis was used to describe the demographics of mothers at baseline, the sources of information used, their perceived helpfulness and the sources used most often at 6 months and 5 years of age. Numbers and percentages are reported. Pearson's *χ* ^2^ tests or Fisher's exact tests (where appropriate) were used to examine the association between the use of health professionals at each age and the use of health professionals over time and mothers' sociodemographic variables.

Multiple logistic regression models were built to identify sociodemographic factors associated with seeking information from health professionals at each age (i.e., answering ‘yes’ to the use of these sources at each age), as well as the factors associated with never using health professionals (i.e., answering ‘no’ to the use of these sources at both ages). All sociodemographic variables with a *p* < 0.25 in the *χ* ^2^ or Fisher's exact tests were entered into the models and nonsignificant predictors were then sequentially removed (starting with the highest *p* value) until only significant predictors remained. To test for confounding, all predictors were then added to the model one by one. As the mothers participated in a health promotion intervention trial, the models were adjusted for intervention group allocation in each trial. All *p* values were two‐sided, with significance set at 0.05.

### 2.5 Ethics statement

The linked trials from which this data was drawn were approved by the Ethics Review Committee of Sydney Local Health District (Protocol No. X16–0360 AND LNR/16/RPAH/495 and Protocol No X18–0387 & HREC/18/RPAH/545).

## 3 RESULTS

When children were 6 months of age, 947 mothers completed the survey (82% response rate) and at 5 years of age, 405 mothers did so (61% response rate). Characteristics of mothers who completed the survey at each time point are shown in Table 1. Approximately half of the mothers were first‐time mothers and almost all were married or in a de facto relationship (i.e., living with a domestic partner on a continuing basis). More than half were over 30 years old, employed, had completed university and had a household income of ≥$80,000 p.a. At both time points, just over 60% of the mothers were born overseas, with ~45% speaking a language other than English at home.

**Table 1:** Demographic characteristics of mothers who completed the 6‐month (*n* = 947) or 5‐year (*n* = 405) surveys.

Demographic characteristics	Completed survey		
	6‐month (*n* = 947) *n* (%)	5‐year (*n* = 405) *n* (%)	Both 6‐month and 5‐year (*n* = 388) *n* (%)
Mothers			
Country of birth			
Australia	361 (38)	151 (37)	147 (38)
Overseas	586 (62)	254 (63)	241 (62)
Language spoken at home			
English	525 (55)	220 (54)	212 (55)
Other	422 (45)	185 (46)	176 (45)
Age			
<30 years	286 (30)	102 (25)	99 (26)
≥30 years	661 (70)	303 (75)	289 (75)
Annual household income^a^			
<$80,000	302 (35)	135 (36)	129 (36)
≥$80,000	551 (65)	237 (64)	226 (64)
Employment status			
Employed	604 (64)	276 (68)	265 (68)
Other	343 (36)	129 (32)	123 (32)
Marital status			
Married/De‐facto partner^b^	887 (94)	384 (95)	367 (95)
Other	59 (6)	20 (5)	20 (5)
Educational attainment			
Up to TAFE/HSC	303 (32)	113 (28)	111 (29)
University	642 (68)	291 (72)	276 (71)
First time mother			
Yes	515 (54)	226 (56)	216 (56)
No	432 (46)	179 (44)	172 (44)
Fathers			
Employment status			
Employed	850 (92)	366 (92)	350 (92)
Other	78 (8)	33 (8)	32 (8)
Educational attainment			
Up to TAFE/HSC	352 (39)	144 (37)	139 (37)
University	556 (61)	251 (64)	239 (63)

Abbreviations: HSC, higher school certificate; TAFE, technical and further education.

^a^ Income reported in Australian dollars.

^b^ A de facto relationship refers to living with a domestic partner on a continuing basis.

Table 2 shows the sources of feeding and nutrition information used by mothers and their perceived helpfulness, at ages 6 months and 5 years. At both time points, friends and family were the most used source of nutrition and feeding information (used by 79% at 6 months and 57% at 5 years) and were perceived as ‘somewhat helpful’ by more than half of the mothers. Websites were also used by many mothers at both ages (used by 63% at 6 months and 48% at 5 years); they were perceived as ‘somewhat helpful’ by more than half of mothers. At 6 months, almost three‐quarters of mothers (74%) sought infant feeding advice from CFHNs; by 5 years, less than one‐fifth of mothers (19%) were seeking advice from CFHNs about their child's food and drink choices. Similarly, a larger proportion (61%) of mothers engaged with other health professionals at 6 months, than at 5 years (9%–37%, dependent on profession). At both ages, health professionals were perceived as a ‘very helpful’ source of information by more than half of mothers seeking their advice.

**Table 2:** Sources of feeding and nutrition information, their perceived usefulness and sources of information used most often when children were aged 6 months and 5 years old.

Source of information (multiple responses allowed)	Use, *n* (%)	Perceived helpfulness, *n* (%)	Source of information used most often, *n* (%)
Age 6 months (*n* = 947)			
Friends and family	746 (79)	Very helpful = 269 (36)	250 (27)
		Somewhat helpful = 452 (61)	
		Not helpful at all = 25 (3)	
Child and family health nurse	701 (74)	Very helpful = 431 (62)	187 (20)
		Somewhat helpful = 245 (35)	
		Not helpful at all = 25 (4)	
Other health professional	577 (61)	Very helpful = 343 (59)	165 (18)
		Somewhat helpful = 220 (38)	
		Not helpful at all = 14 (2)	
Child Blue Book^a^	433 (46)	Very helpful = 201 (46)	15 (2)
		Somewhat helpful = 224 (52)	
		Not helpful at all = 8 (2)	
Telephone helpline	309 (33)	Very helpful = 183 (59)	31 (3)
		Somewhat helpful = 110 (36)	
		Not helpful at all = 16 (5)	
Social media/blogs	465 (49)	Very helpful = 156 (34)	44 (5)
		Somewhat helpful = 291 (63)	
		Not helpful at all = 18 (4)	
Apps	289 (31)	Very helpful = 127 (44)	23 (3)
		Somewhat helpful = 154 (53)	
		Not helpful at all = 8 (3)	
Websites	596 (63)	Very helpful = 250 (42)	150 (16)
		Somewhat helpful = 334 (56)	
		Not helpful at all = 12 (2)	
Age 5 years (*n* = 405)			
Friends and family	229 (57)	Very helpful = 97 (42)	116 (34)
		Somewhat helpful = 128 (56)	
		Not helpful at all = 4 (2)	
Child and family health nurse	78 (19)	Very helpful = 52 (67)	11 (3)
		Somewhat helpful = 26 (33)	
		Not helpful at all = 0 (0)	
General practitioner	151 (37)	Very helpful = 95 (63)	50 (15)
		Somewhat helpful = 55 (36)	
		Not helpful at all = 1 (1)	
Paediatrician	38 (9)	Very helpful = 22 (58)	6 (2)
		Somewhat helpful = 14 (37)	
		Not helpful at all = 2 (5)	
Dietitian	36 (9)	Very helpful = 22 (61)	8 (2)
		Somewhat helpful = 13 (36)	
		Not helpful at all = 1 (3)	
Other health professional	39 (10)	Very helpful = 22 (56)	8 (2)
		Somewhat helpful = 13 (33)	
		Not helpful at all = 4 (10)	
Educators	104 (26)	Very helpful = 53 (51)	16 (5)
		Somewhat helpful = 50 (48)	
		Not helpful at all = 1 (1)	
Social media/blog	149 (37)	Very helpful = 58 (39)	37 (11)
		Somewhat helpful = 87 (58)	
		Not helpful at all = 4 (3)	
Websites	194 (48)	Very helpful = 79 (41)	91 (27)
		Somewhat helpful = 113 (58)	
		Not helpful at all = 2 (1)	

^a^ The Blue Book is a personal health record book provided to parents of every child born in New South Wales, Australia.

### 3.1 Sociodemographic characteristics and the use of health professionals

Table 3 shows that at 6 months, multiparous mothers were less likely to seek advice about breastfeeding or formula feeding from CFHNs (adjusted odds ratio [AOR]: 0.558, 95% confidence interval [95% CI]: 0.416–0.749, *p* < 0.001) and other health professionals (AOR: 0.706, 95% CI: 0.542–0.919, *p* = 0.01). Mothers who were not married or in a de facto relationship were less likely to seek advice from other health professionals (AOR: 0.582, 95% CI: 0.342–0.990, *p* = 0.046) (Table 3).

**Table 3:** Demographic characteristics associated with use of CFHNs and other health professionals for child feeding and nutrition information.

	Age 6 months						
		Using CFHN			Using other health professionals^a^		
Variables	Total *N* = 947 *n* (%)	*n* (row %)	AOR (95% CI)	*p*	*n* (row %)	AOR (95% CI)	*p*
First time mother							
First time mother	515 (54)	408 (79)	Ref		332 (65)	Ref	
Not first time mother	432 (46)	293 (68)	0.558 (0.416–0.749)	<0.001	244 (56)	0.706 (0.542–0.919)	0.01
Marital status							
Married/de facto^b^	887 (94)				547 (62)	Ref	
Other	59 (6)				29 (49)	0.582 (0.342–0.990)	0.046

	Age 5 years						
	Total *N* = 405 *n* (%)	*n* (row %)	AOR (95% CI)	*p*	*n* (row %)	AOR (95% CI)	*p*
Annual household income^c^							
<$80,000	135 (36)	38 (28)	Ref				
≥$80,000	237 (64)	37 (16)	0.514 (0.302–0.875)	0.014			
Country of birth							
Overseas	254 (63)	57 (22)	Ref				
Australia	151 (37)	18 (12)	0.462 (0.257–0.833)	0.010			
First time mother							
First time mother	226 (56)				16 (7)	Ref	
Not first time mother	179 (44)				23 (13)	2.008 (1.021–3.949)	0.043

Abbreviations: AOR, adjusted odds ratio (odds ratio adjusted for intervention group allocation and other sociodemographic variables listed in the table); CFHNs, child and family health nurses; CI, confidence interval; Ref, reference group for analysis.

^a^ In this analysis, only the use of ‘other health professionals’ not further described is presented (i.e., at 5 years this does not include reported use of general practitioners, paediatricians or dietitians).

^b^ A de facto relationship refers to living with a domestic partner on a continuing basis.

^c^ Income reported in Australian dollars.

**Table 4:** Demographic characteristics associated with never using information from CFHNs or other health professionals at both 6 months and 5 years of age.

Variables	Total *N* = 388 *n* (%)	Never used CFHN			Never used other health professionals^a^		
		*n* (row %)	AOR (95% CI)	*p*	*n* (row %)	AOR (95% CI)	*p*
First time mother							
First time mothers	216 (56)	34 (16)	Ref		38 (18)	Ref	
Not first time mothers	172 (44)	49 (28)	2.114 (1.272–3.516)	0.004	45 (26)	1.831 (1.105–3.305)	0.019
Fathers' educational attainment							
University	239 (63)	41 (17)	Ref				
Up to TAFE/HSC	139 (37)	42 (30)	2.081 (1.256–3.449)	0.004			
Mothers' educational attainment							
University	276 (71)				53 (19)	Ref	
Up to TAFE/HSC	111 (29)				30 (27)	1.769 (1.025–3.051)	0.040
Mothers' employment status							
Other	123 (32)				20 (16)	Ref	
Employed	265 (68)				63 (24)	2.058 (1.135–3.733)	0.017

Abbreviations: AOR, adjusted odds ratio (odds ratio adjusted for intervention group allocation and other sociodemographic variables listed in the table); CFHNs, Child and family health nurses; CI, confidence interval; HSC, higher school certificate; Ref, reference group for analysis; TAFE, technical and further education.

^a^ In this analysis, the other health professionals at 6 months included the category ‘other health professionals’ not further described; at 5 years, it included use of general practitioners, paediatricians, dietitians, or other health professionals not further described.

At 5 years, mothers with household income ≥$80,000 p.a. (AOR: 0.514, 95% CI: 0.302–0.875, *p* = 0.014) and those born in Australia (AOR: 0.462, 95% CI: 0.257–0.833, *p* = 0.01) were less likely to seek information about food and drink choices for their child from a CFHN. Mothers born in Australia (AOR: 0.431, 95% CI: 0.274–0.677, *p* < 0.001) and those who were employed (AOR: 0.581, 95% CI: 0.374–0.905, *p* = 0.016) were less likely to seek advice from their GP. Multiparous mothers were less likely to seek advice from a paediatrician (AOR: 0.439, 95% CI: 0.206–0.936, *p* = 0.033) and more likely to seek advice from ‘other health professionals’ (AOR: 2.008, 95% CI: 1.021–3.949, *p* = 0.043). There were no significant sociodemographic predictors of engagement with dietitians at 5 years of age (Table 3).

### 3.2 Sociodemographic characteristics associated with never seeking advice from health professionals

Multiparous mothers (AOR: 2.114, 95% CI: 1.272–3.516, *p* = 0.004) and mothers of children whose fathers had not attended university (AOR: 2.081, 95% CI: 1.256–3.449, *p* = 0.004) were more likely to have never sought nutrition or feeding advice from a CFHN. Mothers who had not attended university (AOR: 1.769, 95% CI: 1.025–3.051, *p* = 0.040), multiparous mothers (AOR: 1.831, 95% CI: 1.105–3.035, *p* = 0.019) and those who were employed (AOR: 2.058, 95% CI: 1.135–3.733, *p* = 0.017) were more likely to have never sought advice from other health professionals (Table 4).

## 4 DISCUSSION

This secondary analysis of two cross‐sectional surveys found that sociodemographic factors were associated with mothers' engagement with health professionals for infant and child feeding and nutrition advice. Multiparous and unmarried mothers were less likely to seek advice from health professionals at 6 months of age, whereas mothers born overseas were more likely to engage with both CFHNs and GPs at 5 years of age for advice regarding children's food and drink choices. Multiparous mothers, families with lower levels of parental education and working mothers were less likely to seek infant and child feeding advice from health professionals at all. The results also show a decline in information seeking from health professionals from 6 months to 5 years of age, with 74% of mothers having sought advice about breast and formula feeding from CFHNs at 6 months, compared with <20% seeking advice about food and drink choices for their child from CFHNs at 5 years. In comparison, friends and family, and websites remained popular sources of infant and child feeding and nutrition information at both ages.

### 4.1 Sociodemographic factors associated with seeking advice from health professionals as children age

In this study, a decline in the use of information about child feeding and nutrition information from health professionals at 5 years compared with 6 months was observed. This is consistent with the results of a survey of CFHNs around Australia, which found that when the proportion of CFHNs providing support to children weekly was broken down by age group, support declined as children got older (Schmied et al., 2014). Although there is a recognized decline in child and family health service use as children get older, it is also important to recognize that data collection at 6 months occurred before the COVID‐19 pandemic, while 5‐year data collection commenced 6 months after the end of the final COVID‐19 lockdown in NSW, Australia. The COVID‐19 pandemic impacted health service delivery worldwide. In NSW, where this study took place, child and family health services were directed to prioritize face‐to‐face care for high‐risk families, with many other services moved to telehealth (NSW COVID‐19 Clinical Council, 2022). It is likely that during the period of 5‐year data collection, service delivery was in the process of returning to normal operations and face‐to‐face contact with health professionals may have remained limited which could have impacted parental capacity to seek preventive health care during this time.

In this study, mothers born overseas were more likely to seek advice from CFHNs and GPs at 5 years of age. This stands in contrast to previous literature which has reported on the barriers that individuals from culturally and linguistically diverse backgrounds may experience in accessing healthcare in Australia, including a lack of information about the services available, challenges with accessing and communicating with health professionals, as well as cultural differences in conceptions of health and illness (Khatri & Assefa, 2022). Previous research has identified that, of families from culturally and linguistically diverse backgrounds, those who are most at risk of missing out on early childhood developmental surveillance are those with lower levels of English proficiency, lower maternal education levels and lower socioeconomic status (Woolfenden et al., 2015). Given that English proficiency was a requirement of study participation, it is possible that the most vulnerable families from culturally and linguistically diverse backgrounds were not included in the trials, and thus the lower heath service usage seen in previous research was not reflected in this work.

### 4.2 Factors associated with never seeking advice from health professionals

Our research highlights three key factors that may impact engagement with health professionals for infant and child feeding and nutrition advice regardless of child age—parity, parental education level and maternal employment. The finding that multiparous mothers were less likely to seek any infant and child feeding advice from health professionals may reflect lower engagement of multiparous women with child health services in general. International studies have found that multiparous mothers were less engaged with child nurses and parents groups than primiparous mothers and that first‐time mothers were more likely to report experiencing challenges with infant and young child feeding which may prompt more help‐seeking behaviour (De Rosso et al., 2022; Lagerberg & Magnusson, 2013). Qualitative research from Australia may provide some explanation as to the lower engagement of multiparous women with health professionals, as mothers reported that with subsequent children their confidence in managing child feeding increased and they were more comfortable balancing their own experience against the advice received from health professionals (Alexander et al., 2015).

The ability to engage meaningfully with, and seek information from, health professionals is an important component of functional health literacy (Bo et al., 2014; Nutbeam, 2000). Previous research highlights a positive relationship between educational attainment and health literacy, which may influence access to and use of information from health professionals (Barber et al., 2009; Martin et al., 2009). Thus, it may be posited that the results of this study, showing an association between lower levels of parental education and a higher likelihood of not seeking advice from health professionals, may be influenced by health literacy. Parents with lower health literacy may feel less confident recognizing when preventive care may be warranted and be less comfortable seeking advice from health professionals and navigating the healthcare system (Paasche‐Orlow & Wolf, 2007). However, a health literacy lens may not fully explain our findings as maternal education influenced mothers' engagement with GPs, but only fathers' education level, influenced their engagement with CFHNs.

The finding that maternal employment was associated with a greater likelihood of never seeking infant and child feeding advice from health professionals may be associated with reduced time to access preventive health services. This would be consistent with findings from studies in the United States, which found that, as a mothers working hours increase, the likelihood of completing recommended preventive care visits decreases (Hamman, 2011; Shepherd‐Banigan et al., 2017). However, these studies note that access to paid leave mediates the impact of maternal employment on access to preventive care, data that were not collected as part of this study.

### 4.3 Preferred sources of infant and child feeding and nutrition information

The results of this study indicate that family and friends and websites are both important sources of information about infant and child feeding and nutrition that parents continued to engage with at 5 years of age. This is consistent with the findings of previous research in Australia and the United States, which have found that a variety of information sources influence mothers' infant feeding practices, with peers and the internet highlighted as important sources (Spence et al., 2016), and mothers reporting that they valued hearing about others personal experiences of different infant feeding practices (Weber et al., 2023). Given this preference to learn from the experiences of others, this research points to opportunities to encourage peer learning that is guided by evidence‐based sources, for example, qualitative research in NSW found that CFHN‐led new parent groups were valued by mothers as a space to learn from each other and seek reassurance about their parenting practices (Guest & Keatinge, 2009).

With websites being a popular source of information about infant and child feeding, it is important that the information available to parents reflects best practices. A 2022 review of websites providing information about health behaviours during infancy, found that the overall scope and depth of the information provided was generally poor, with the majority of websites not catering for users with lower literacy levels and not providing culturally appropriate information for their intended audiences (Jawad et al., 2022). Given that many mothers report seeking infant and child feeding information online, this points to opportunities to improve the quality of the information available to parents in this format.

There is a substantial body of literature examining the impacts upon early childhood nutrition in the first 2 years of life. This study extends upon the existing literature using longitudinal data with repeated surveys up until children reached 5 years of age, providing insights into the changing influences on infant and child feeding throughout early childhood. The data included in this study came from mothers who were enrolled in RCTs with a focus on early childhood obesity prevention. As such, it may have captured a group of interested mothers and, thus, the results may not be reflective of all mothers living in Australia, whose knowledge of the health system and sources of advice may vary. Furthermore, the survey items in this secondary analysis explored specific aspects of infant feeding and child nutrition (i.e., breast and formula feeding at 6 months, and food and drink choices at 5 years), and the results may not be applicable to other feeding practices. In particular, a national survey in Australia, where this study was conducted, found that many parents do not follow complementary feeding guidelines to introduce solid foods around 6 months of age, with more than a quarter of parents introducing solids before 6 months of age, and a small proportion delaying the introduction of solids beyond 7 months (Netting et al., 2022). Given that parents report health professionals to be an important source of information regarding complementary feeding (Spyreli et al., 2021), further research should examine factors that may impact parental engagement with health professionals for complementary feeding advice. Further qualitative research is also needed to understand the factors that influence mothers' choice of information sources and future analyses should consider how reliance on different sources of infant feeding and nutrition information may impact feeding behaviours and dietary intake.

## 5 CONCLUSION

This study highlights that parent use of child and family health services for nutrition and feeding advice declines with age. This has important implications for health service provision in early childhood, as other health professionals, particularly GPs, may be in a favourable position to continue to promote positive feeding behaviours as children reach school age. Our findings also highlight opportunities to engage mothers in discussions of infant and child nutrition and feeding in primary healthcare settings. In particular, flexible service delivery to support working mothers, the provision of nutrition information in formats that are accessible to families with lower health literacy, and proactive discussions of nutrition with multiparous mothers to highlight changes in infant feeding guidelines over time, may help reach mothers who are less likely to seek infant and child feeding advice from health professionals. Given that our study indicates that mothers obtain infant and child feeding and nutrition information in various formats it is also important to ensure evidence‐based information that complements services provided by health professionals is accessible in formats acceptable to mothers, particularly online and through peer learning models.

## AUTHOR CONTRIBUTIONS

Li Ming Wen was the principal investigator of the CHAT studies and contributed to the study design and data collection. All authors contributed to the conception of this secondary analysis. Eve House conducted the analysis with statistical support from Huilan Xu. Eve House drafted the manuscript. Huilan Xu, Sarah Taki, Elizabeth Denney‐Wilson, Louise Baur and Li Ming Wen critically reviewed the manuscript. All authors provided final approval of the version to be published and agree to be accountable for all aspects of the work.

## CONFLICT OF INTEREST STATEMENT

The authors declare no conflict of interest.

## Supporting information

Supporting Information.

## Data Availability

The data that support the findings of this study are available on request from the corresponding author. The data are not publicly available due to privacy or ethical restrictions.
