# Supplementary material for: Mothers' sociodemographic factors and use of health professionals for child feeding advice
Source: Matern Child Nutr. 2023 Nov 6;20(1):e13586. doi: 10.1111/mcn.13586 (PMC10750020; doi:10.1111/mcn.13586)
Supplement: Supplementary file 1 — Supporting Information. [file MCN-20-e13586-s001.docx]

**APPENDIX 1**

**6-month survey items**

[Sources of support, trust in source and utility of information]

65. If you have had questions about breast or formula feeding, we would like to know a bit more about how you found information and what you found to be most useful for making decisions about breast or formula feeding [child first name].

| Answering yes or no, when you had questions about breastfeeding or formula feeding, did you seek out advice or information from the following sources: **OPTIONS [read out]:** | [Only ask follow-up question (below) if the option YES in the left column is selected]  **[Say] When you got this advice, was it** [please select one option only]: |
| --- | --- |
| Friends & family   - Yes – ask question to right → - No – go to next option | - Very helpful - Somewhat helpful - Not helpful at all |
| Child & family nurse   - Yes – ask question to right → - No – go to next option | - Very helpful - Somewhat helpful - Not helpful at all |
| Other health professional   - Yes – ask question to right → - No – go to next option | - Very helpful - Somewhat helpful - Not helpful at all |
| the Child Blue Book   - Yes – ask question to right → - No – go to next option | - Very helpful - Somewhat helpful - Not helpful at all |
| [For intervention groups only]  Healthy Beginnings   - Yes – ask question to right → - No – go to next option | - Very helpful - Somewhat helpful - Not helpful at all |
| Telephone helpline   - Yes – ask question to right → - No – go to next option | - Very helpful - Somewhat helpful - Not helpful at all |
| Social media/ blog   - Yes – ask question to right → - No – go to next option | - Very helpful - Somewhat helpful - Not helpful at all |
| Apps   - Yes – ask question to right → - No – go to next option | - Very helpful - Somewhat helpful - Not helpful at all |
| Websites   - Yes – ask question to right → - No – go to next option | - Very helpful - Somewhat helpful - Not helpful at all |

66. Which of the following sources of information did you use *most frequently* to make decisions about breast and formula feeding? [select only *one* option]:

| **OPTIONS SHOW SELECTIONS MADE IN QUESTION 65 ONLY [read out]:** |
| --- |
| - Friends & family |
| - Child & family nurse |
| - Other health professional |
| - the Child Blue Book |
| - [For intervention groups only] Healthy Beginnings |
| - Telephone helpline |
| - Social media/ blog |
| - Apps |
| - Websites |

**5-year survey items**

48. If you have had questions about the type of foods and drinks to give your child, we would like to know a bit more about how you found information and what you found to be most useful for making decisions about your child’s nutrition.

| Answering yes or no, when you had questions about your child’s food choices, did you seek out advice or information from the following sources: **OPTIONS [read out]:** | [Only ask follow-up question (below) if the option YES in the left column is selected]  **[Say] When you got this advice, was it** [please select *one* option only]: |
| --- | --- |
| Friends & family   - Yes – ask question to right → - No – go to next option | - Very helpful - Somewhat helpful - Not helpful at all |
| Child & family nurse   - Yes – ask question to right → - No – go to next option | - Very helpful - Somewhat helpful - Not helpful at all |
| General Practitioner   - Yes – ask question to right → - No – go to next option | - Very helpful - Somewhat helpful - Not helpful at all |
| Paediatrician   - Yes – ask question to right → - No – go to next option | - Very helpful - Somewhat helpful - Not helpful at all |
| Dietitian   - Yes – ask question to right → - No – go to next option | - Very helpful - Somewhat helpful - Not helpful at all |
| Other health professionals   - Yes – ask question to right → - No – go to next option | - Very helpful - Somewhat helpful - Not helpful at all |
| Educators (e.g. childcare or school teachers)   - Yes – ask question to right → - No – go to next option | - Very helpful - Somewhat helpful - Not helpful at all |
| Social media/ blog   - Yes – ask question to right → - No – go to next option | - Very helpful - Somewhat helpful - Not helpful at all |
| Websites   - Yes – ask question to right → - No – go to next option | - Very helpful - Somewhat helpful - Not helpful at all |

49. Which of the following sources of information did you use *most frequently* to make decisions about the food and drinks you provide for your child? [select only *one* option]:

| **OPTIONS**  **SHOW SELECTIONS MADE IN QUESTION 48 ONLY**  **[read out]:** |
| --- |
| - Friends & family |
| - Child & family nurse |
| - General Practitioner |
| - Paediatrician |
| - Dietitian |
| - Other health professionals |
| - Educators (e.g. childcare or school teachers) |
| - Social media/ blog |
| - Websites |
